# Compartmentalized and Differential Regulation of NK- and γδ T-Cell Degranulation by Trophoblast-Derived Signals During Early Pregnancy

**DOI:** 10.3390/cells15181691

**Published:** 2026-09-17

**Authors:** Mariela Ivanova, Marina Alexandrova, Dimitar Parvanov, Diana Manchorova, Antonia Terzieva, Ivaylo Vangelov, Kiril Kirilov, Tanya Dimova

**Affiliations:** 1 Institute of Biology and Immunology of Reproduction, Bulgarian Academy of Sciences, 1113 Sofia, Bulgaria; mariela.ivanova021@gmail.com (M.I.); marina.m.alexandrova@gmail.com (M.A.); anterzieva@abv.bg (A.T.); vangelovivaylo@gmail.com (I.V.); 2 Nadezhda Women’s Health Hospital, 1330 Sofia, Bulgaria; dimparvanov@abv.bg; 3 C.S. Mott Center for Human Growth and Development, Wayne State University, Detroit, MI 48202, USA; diana_man4orova@abv.bg; 4 Institute of Molecular Biology, Bulgarian Academy of Sciences, 1113 Sofia, Bulgaria; kkirilov@bio21.bas.bg

**Keywords:** early human pregnancy, maternal–fetal interface, γδ T cells, NK cells, degranulation, extravillous trophoblast, HLA

## Abstract

**Highlights:**

**What are the main findings?**

- During early human pregnancy γδ T cells display greater spontaneous degranulation than NK cells. At baseline, decidual NK and γδ T cells degranulate more than paired blood-derived counterparts.
- Pregnancy-adapted NK cells show limited responsiveness to trophoblast-derived signals, whereas decidual CD56+ γδ T cells remain responsive.

**What are the implications of the main findings?**

- Triggered degranulation of decidual CD56+ γδT cells against extravillous trophoblasts with altered HLA patterns indicates their high sensitivity to local shifts in trophoblast immune signaling.
- Understanding how decidual NK and γδT cells are regulated may help explain immune (in)tolerance and surveillance underlying pregnancy complications.

**Abstract:**

Cytotoxic lymphocytes contribute to immune regulation at the maternal–fetal interface, yet their regulating mechanisms during early pregnancy remain elusive. We investigated compartment-specific NK- and γδT-cell degranulation towards trophoblast-derived signals. Blood and decidual mononuclear cells were obtained from non-pregnant women and women in early pregnancy. Spontaneous and trophoblast-mediated degranulation of NK- and γδT-cell subsets was assessed following co-culture with Sw71 extravillous trophoblast-like cells with normal and reduced expression of HLA-G/HLA-C molecules. CD107a-positive frequency and mean fluorescence intensity were quantified by flow cytometry. Statistical analyses included paired testing, multivariate analyses, and false discovery rate correction. Decidual cytotoxic lymphocytes displayed a distinct degranulation profile compared with blood counterparts. At baseline, γδT cells exhibited higher spontaneous degranulation than NK cells. Trophoblast co-culture exerted limited effects on NK-cell degranulation; however, decidual CD56^+^ γδ T cells showed increased degranulation towards trophoblasts with impaired HLA expression. In conclusion, early pregnancy is characterized by a highly compartmentalized regulation of cytotoxic lymphocyte activity. Decidual NK and γδT cells exhibit enhanced degranulation, but only CD56^+^ γδT cells retain substantial responsiveness to trophoblast-derived signals. These findings identify γδT cells as a dynamically regulated cytotoxic population at the maternal–fetal interface, sensitive to local shifts in trophoblast immune signaling.

## 1. Introduction

**Figure 1:**
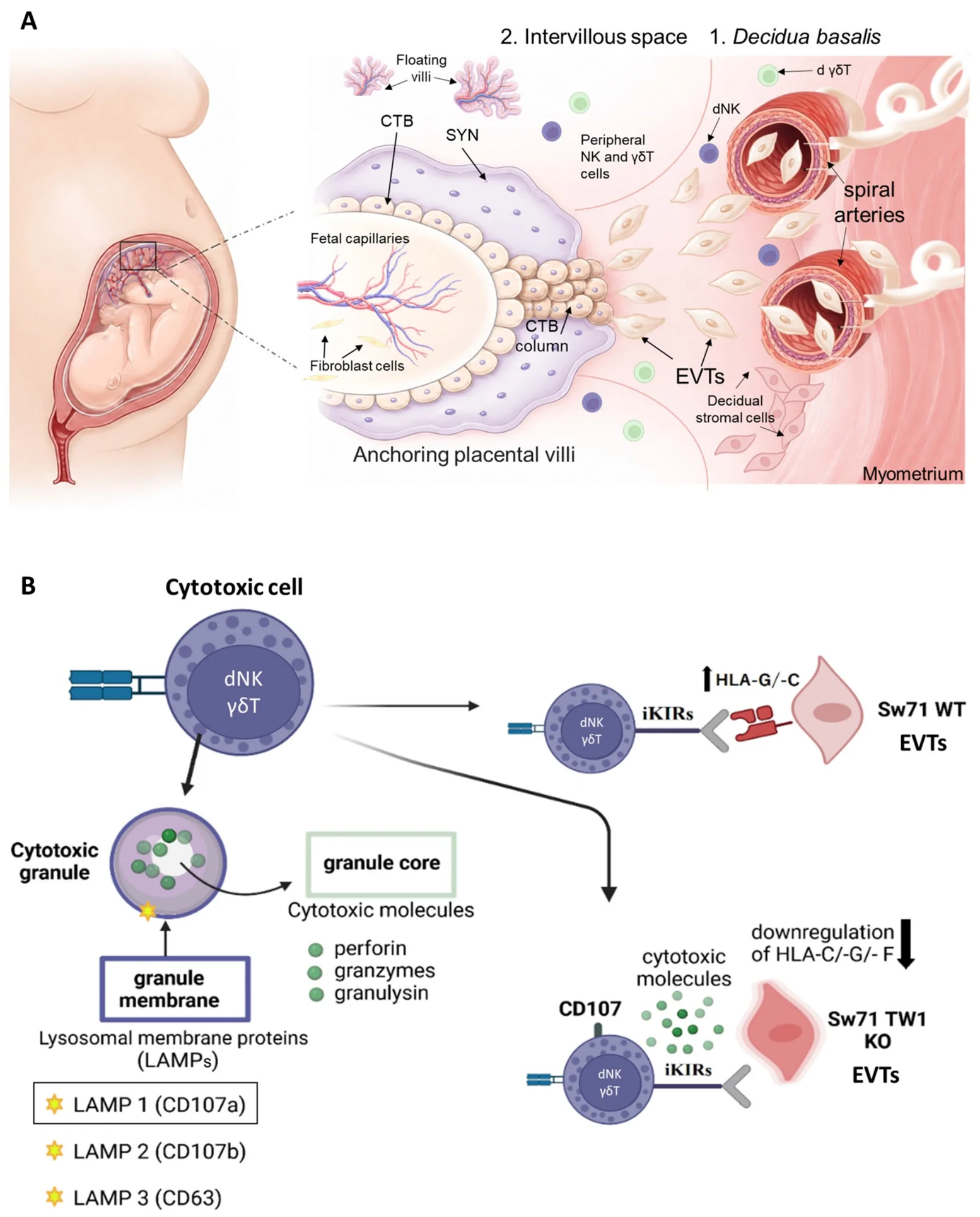
Maternal–fetal interface in human early pregnancy (**A**) and hypothetical model of regulated degranulation of cytotoxic NK or γδ T cells upon interaction with EVTs (**B**). (**A**)—Schematic view of the maternal–fetal interface and two contact zones of the fetal-derived trophoblasts with the maternal immune cells: (1) in the decidua basalis, where EVTs make contact with local maternal immune cells, and (2) in the intervillous space, where syncytiotrophoblasts are in contact with the peripheral blood maternal immune cells. (**B**)—Degranulation of cytotoxic cells (NK or γδ T cells) upon interaction with EVTs showing normal or reduced expression of HLA molecules. NK cells and γδ T cells contain cytotoxic granules (perforin, granzyme A, granzyme B, granulysin) and express iKIRs, recognizing HLA-G and HLA-C molecules on the surface of the EVTs. Upon co-culture with EVTs showing normal HLA-G and HLA-C expression (Sw71 cells) iKIR/HLA-G and iKIR/HLA-C interactions would lead to blocking of NK- or γδ T-cell degranulation. In contrast, reduced expression of these HLA molecules on the EVTs (Sw71 *TW1*-KO cells) would not substantially engage iKIRs, resulting in priming of activatory KIRs, provoking increased degranulation of the cytotoxic cells. CTB—cytotrophoblast; SYN—syncytiotrophoblast; EVTs—extravillous trophoblasts. Figure 1 was created with Medical Illustrative Tools.

Successful human pregnancy requires the establishment of immune tolerance toward a semi-allogeneic fetus while preserving the capacity of the maternal immune system to respond to pathogens and malignant transformation [1,2]. Rather than being globally suppressed, the maternal immune system undergoes extensive functional adaptation that supports implantation, placentation, and fetal development while maintaining immune competence. These processes are particularly important during early pregnancy, when embryo-derived trophoblast cells establish intimate contact with maternal decidual tissues and immune cells at the maternal–fetal interface. A defining feature of human pregnancy is the deep invasion of extravillous trophoblasts (EVTs) into the decidua basalis [3]. EVTs migrate through maternal tissues, participate in spiral artery remodeling, and establish direct interactions with decidual leukocytes, thereby contributing to the development of a local immune environment permissive for fetal growth [4,5]. The maternal–fetal interface, therefore, represents a highly specialized immunological niche in which maternal immune cells continuously interact with fetal-derived trophoblasts in two contact zones: (1) in the decidua basalis, where EVTs make contact with local maternal immune cells, and (2) in the intervillous spaces, where syncytiotrophoblasts covering the placental villi (villous trophoblasts) are in contact with the peripheral blood maternal immune cells (Figure 1). Successful implantation and placentation depend on the precise regulation of trophoblast–immune-cell interactions, as disturbances in immune adaptation have been associated with implantation failure, recurrent pregnancy loss, preeclampsia, and other pregnancy complications. Among the leukocyte populations present at the maternal–fetal interface, natural killer (NK) cells and γδ T cells are of particular interest because of their cytotoxic potential and ability to recognize cellular stress independently of classical antigen presentation. Decidual NK (dNK) cells constitute up to 70% of decidual leukocytes during the first trimester and are predominantly represented by the CD56^bright^ subset [6,7]. Although dNK cells possess cytotoxic granules containing perforin, granzymes, and granulysin, they are generally considered functionally distinct from peripheral blood NK cells and are thought to contribute to trophoblast invasion, vascular remodeling, and placental development through the production of cytokines and angiogenic factors [8,9]. γδ T cells are also enriched in early pregnancy decidua compared with peripheral blood and represent an important component of local immune surveillance [10,11,12]. These cells combine characteristics of innate and adaptive immunity, expressing a T-cell receptor while simultaneously utilizing several NK-associated receptors to recognize stress-induced ligands, transformed cells, and infected targets [13,14]. During early pregnancy the decidual γδ T cells display an activated and differentiated phenotype and contain the whole range of cytotoxic granules [11,15]. Despite the presence of abundant cytotoxic lymphocytes at the maternal–fetal interface, healthy trophoblasts are normally protected from immune-mediated destruction. This state of controlled immune tolerance is achieved through multiple regulatory mechanisms, including the unique expression pattern of major histocompatibility complex (MHC) class I molecules by EVTs. Unlike most somatic cells, EVTs express the non-classical HLA class I molecules HLA-G and HLA-E together with HLA-C, the only polymorphic classical HLA class I molecule [16,17]. These molecules interact with inhibitory receptors expressed by decidual immune cells, particularly killer-cell immunoglobulin-like receptors (KIRs) and CD94/NKG2A, thereby contributing to the maintenance of fetal tolerance [18,19]. Both NK and γδ T cells express KIRs capable of recognizing trophoblast-associated HLA molecules [13,20,21]. Importantly, tolerance at the maternal–fetal interface does not imply the absence of cytotoxic potential. Decidual NK and γδ T cells retain the molecular machinery required for cytotoxic responses, including perforin-, granzyme-, and granulysin-containing granules [15,22]. Under physiological conditions, however, these cells exhibit tightly controlled effector functions, allowing them to participate in tissue remodeling and immune surveillance without damaging healthy trophoblasts [23,24]. Cytotoxic degranulation represents a key functional mechanism through which NK and γδ T cells exert their effector activity, and therefore provides an informative measure of their activation state and responsiveness to local regulatory signals. By releasing cytolytic granules, they can induce apoptosis of trophoblast cells, and, when excessive or dysregulated, this can lead to pregnancy complications [25]. It has been noted that during early human pregnancy decidual NK and γδ T cells express inhibitory KIRs (iKIRs) [20,21]. Moreover, the absence or insufficiency of HLA-G or HLA-C ligands for the iKIRs may disrupt maternal dNK-mediated tolerance towards trophoblasts, leading to adverse pregnancy outcomes [26]. Although considerable progress has been made in understanding decidual immune-cell phenotypes and trophoblast–NK-cell immune interactions [17,27], relatively little is known about how trophoblast-derived signals regulate the cytotoxicity of γδ T-cell subsets in different maternal compartments during early pregnancy. In particular, the contribution of trophoblast HLA-G and HLA-C expression to the modulation of cytotoxic responses in decidual and peripheral immune cells remains incompletely understood. Here, we hypothesized that trophoblast-derived HLA-dependent signals might differentially regulate NK- and γδ T-cell degranulation via inhibitory KIRs (Figure 1). NK cells and γδ T cells contain cytotoxic granules such as perforin, granzymes and granulysin and express iKIRs, recognizing HLA-G and HLA-C molecules on the surface of the EVTs. To check our hypothesis, in this study we used as a model Sw71 EVT-like cells [28] showing optimal HLA-G [29] and HLA-C expression [30] and their *TWIST1* knocked-out derivative, Sw71 *TW1*-KO cells with reduced HLA-G and HLA-C expression [29]. Functionally, Sw71 cells resemble EVTs, showing a hybrid cytokeratin7/vimentin phenotype and the specific HLA-G+/HLA-C+ profile, as well as invasion and migration capacity [28,31]. Upon co-culture with Sw71 EVT-like cells, iKIR/HLA-G and iKIR/HLA-C interactions might control NK- or γδ T-cell degranulation. In contrast, reduced expression of the HLA molecules on the Sw71 *TW1*-KO cells might not substantially engage iKIRs, resulting in priming of activatory KIRs and increased degranulation of the cytotoxic cells.

The aim of the present study was to evaluate site-specific NK- and γδ T-cell degranulation during early pregnancy. Using peripheral blood and decidual immune cells together with wild-type and genetically modified EVT-like cells differing in HLA-G and HLA-C expression, we investigated how trophoblast-associated signals influence the degranulation of these two major cytotoxic lymphocyte populations.

## 2. Materials and Methods

### 2.1. Study Populations and Sample Collection

Healthy pregnant women in early pregnancy, undergoing elective termination of pregnancy (6–12 gw, n = 8, EP—early pregnancy), along with healthy non-pregnant women of reproductive age (control group, n = 7, NP—non-pregnant), were enrolled. All women signed a written informed consent form for the use of blood and tissue samples. This study was carried out in accordance with the Declaration of Helsinki and was approved by the Human Research Ethics Committee at the University Obstetrics and Gynecology Hospital “Maichin Dom”, Sofia, Bulgaria (25-95-75/30.12.2021). Blood and decidua samples were processed within one hour after blood and tissue collection.

### 2.2. Isolation of Peripheral Blood (PBMCs) and Decidual Mononuclear Cells (DMCs)

Paired blood and decidual samples were obtained from the early pregnant women, and blood samples from the non-pregnant controls. For the isolation of PBMCs, the blood was collected in a vacuum container with the anticoagulant lithium heparin, and a standard protocol was used. Briefly, blood diluted with sterile PBS (1:1) was overlaid on Ficoll-Paque™ PLUS separation medium (#17144003, Cytiva, Marlborough, MA, USA), and the interphase band of mononuclear cells was collected. After washing with sterile PBS, the cells were counted and assessed for viability with 0.5% Trypan blue staining. For the isolation of DMCs, fresh 1st trimester decidua basalis tissue was collected after careful separation of the trophoblast. DMC isolation was performed by mechanical disruption in sterile PBS (1–5 g tissue/50 mL). The minced tissue was sequentially filtered through a metal sieve and a 70 µm strainer, followed by centrifugation of the suspension at 370× *g*/15 min. The cell pellet was resuspended in sterile PBS, layered on Ficoll-Paque™ PLUS separation medium and centrifuged at 800× *g* for 20 min. The interphase was collected, washed and then assessed for viability using the Trypan blue exclusion test. Mononuclear cells were left overnight in RPMI complete: RPMI 1640 (#11875093, Gibco, Marlborough, MA, USA) supplemented with 10% inactivated fetal bovine serum (FBS), 0.1 mmol/L MEM, 10 mmol/L (#M7145, Sigma, Burlington, MA, USA), HEPES (#H0887, Sigma, Burlington, MA, USA), 1 mmol/L sodium pyruvate (#11360-039, Gibco, Marlborough, MA, USA) and 100 U/mL penicillin/streptomycin (#15140-122, Gibco, Marlborough, MA, USA).

### 2.3. Cell Lines and Culture Media

Sw71 cells (Cellosaurus Swan71 CVCL_D855) are healthy, non-cancerous trophoblast cells, isolated from normal placenta at 7 gw and immortalized by ectopic expression of the catalytic subunit of telomerase (hTERT) [28]. The cell line is of the adhesive type with a fibroblast-like morphology. The Sw71 TWIST1-KO (Sw71 TW1-KO) cell clone was established by knocking out of the TWIST1 gene using CRISPR/Cas9 technology [29]. The TWIST1 gene encodes the TWIST1 transcriptional factor, involved in the epithelial–mesenchymal transition (EMT) and invasion of EVT cells [32]. Previous characterization demonstrated reduced expression of trophoblast-associated immune regulatory molecules, including HLA-G and HLA-C, in TW1-KO cells [29,33]. Both Sw71 and Sw71 TW1-KO cells were propagated in DMEM-F12 (D8062, Sigma-Aldrich, St. Louis, MO, USA) supplemented with 10% inactivated FBS, 0.1 mmol/L MEM, 10 mmol/L (M7145, Sigma), HEPES (H0887, Sigma), 1 mmol/L sodium pyruvate (11360-039, Gibco) and 100 U/mL penicillin/streptomycin (15140-122, Gibco).

### 2.4. Co-Culture/Degranulation Assay

The degranulation rate of the cytotoxic cells can be easily measured by the detection of the lysosomal CD107a (LAMP-1) molecules on their surface by FACS. CD107 is localized in the membrane of the cytotoxic granules, and upon their exocytosis during degranulation it becomes exposed on the surface of the cytotoxic cells [34,35]. In the present study, surface CD107a expression was used as a functional readout of cytotoxic lymphocyte degranulation. This assay reflects degranulation activity but does not directly measure target-cell death or killing. For the degranulation measurement we co-cultured either Sw71 wild-type (WT) cells or Sw71 *TW1*-KO cells with PBMCs or DMCs from women in early pregnancy (EP, paired samples) or with PBMCs from non-pregnant women (NP). The viability of cell suspensions was monitored using Trypan blue staining before the degranulation assay to be sure that more than 80% of the cells were vital. Cells were co-cultured at an effector:target (E:T) ratio of 1:1 in 1 mL medium (50% DMEM-F12 complete and 50% RPMI complete) supplemented with 0.7 μg/mL Monensin (#M5273, Sigma-Aldrich, St. Louis, MO, USA) following the classical protocol for degranulation assays [36]. During the 6 h incubation at 37 °C/5% CO_2_, 5 μL of anti-CD107a-PE antibody (clone H4A3, #555801, BD, Taufkirchen, Germany) was added.

### 2.5. Flow Cytometry

**Figure 2:**
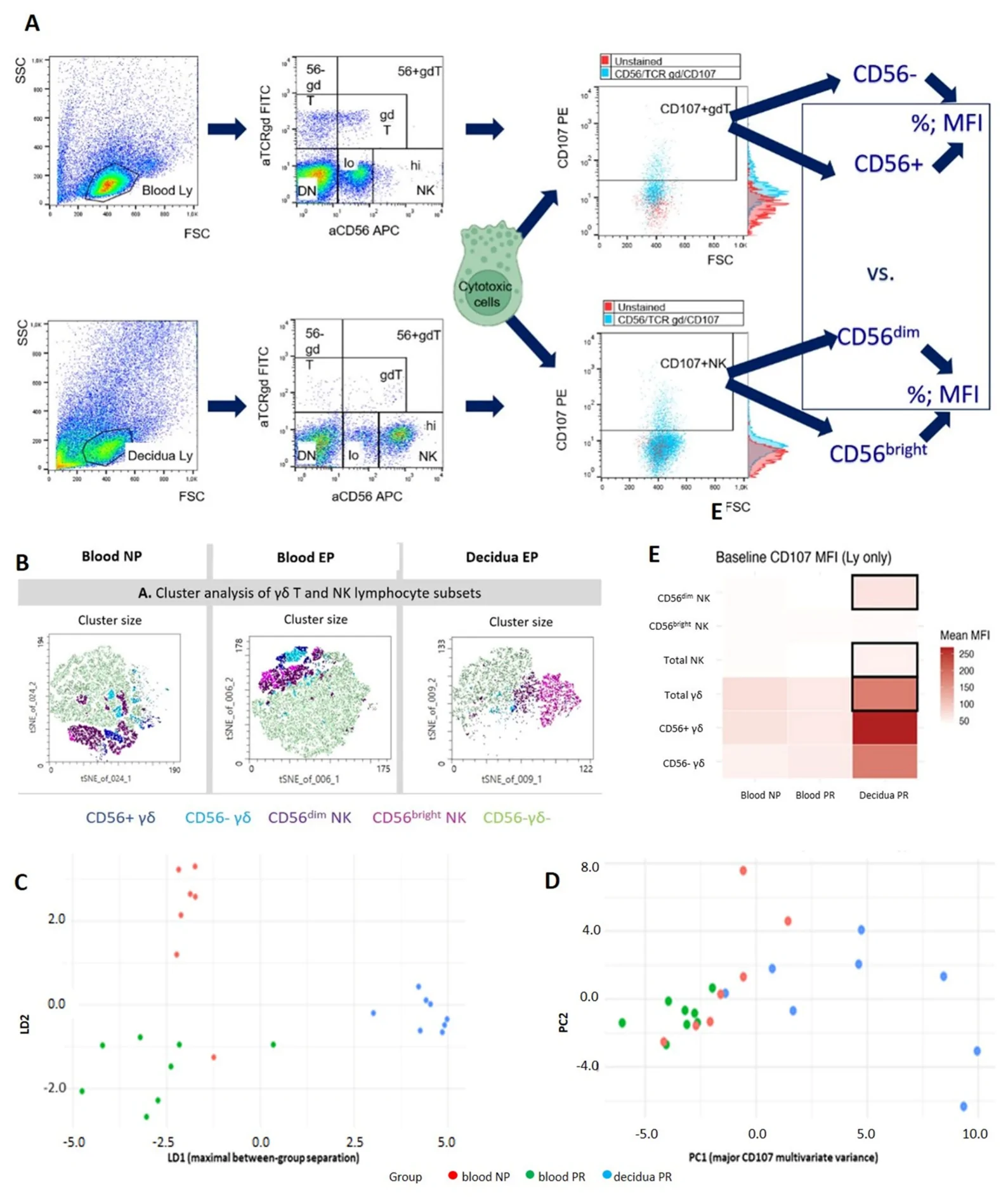
Distribution of NK- and γδ T-cell subsets and their CD107 expression in blood and decidua of pregnant and non-pregnant women. (**A**)—Gate strategy for CD107 detection (representative FACS plots). The lymphocyte gate was placed using FSC vs. SSC plotting, defining either blood (up) or decidual (down) lymphocytes, and confirmed. The suspension was labeled with antibodies against CD56 and γδ T to distinguish the following populations and subsets: γδ T (TCR γδ+) lymphocytes with their CD56- and CD56+ subsets, and NK (CD56+, TCRγδ-) cells with their CD56^dim^ and CD56^bright^ subsets. The CD107a expression (blue) was defined in each population and subset against the unstained control (red) and the MFI and percentage of CD107-positive cells were determined. (**B**)—tSNE-clustered γδ T- and NK-cell subsets in PBMCs of non-pregnant (NP) and pregnant (PR) women as well as in DMCs of pregnant (PR) women. (**C**)—LDA shows a three-way separation: blood (NP): high LD2, moderate LD1; blood (PR): low LD2, moderate LD1; decidua (PR): high LD1, moderate LD2. (**D**)—Clear clustering of decidua samples, revealing a distinct degranulation-associated phenotype compared to blood-derived groups shown by PCA of multivariate CD107 features. (**E**)—Heatmap of mean baseline CD107 MFI per subset across the three groups. Highlighted regions (outlined in black on figure): decidual NK^dim^, NK, and γδ T subsets show markedly elevated baseline CD107 MFI. Peripheral (NP and PR) subsets have uniformly low MFI.

FACS staining of the antigens of interest was performed on collected single or co-cultured mononuclear cells with the following antibodies, CD56-APC (clone MEM-188, Exbio, Praha, a.s., Vestec, Czech Republic), γδ TCR-FITC (clone 11F2, BD, San Jose, CA, USA) and CD107-PE (degranulation assay), or on single EVT-like cells with HLA-C-PE (sc-166088, Santa Cruz Biotechnology, Santa Cruz, CA, USA). Cell suspensions were adjusted to 1 × 10^6^ cells per tube (12 × 75 mm) and washed with 3 mL FACS buffer 0.1% BSA/PBS (#11924, Bovine Serum Albumin, Heidelberg, Germany) and incubated with the antibodies for 20 min at 4 °C, in the dark. After washing, the cells were fixed in 250 μL of 0.5% paraformaldehyde/PBS. Unstained cells and single-stained controls were included for gating and compensation. Cells were collected on a FACSCalibur™ Flow Cytometer (BD Biosciences, San Jose, CA, USA) and analyzed using FlowJo software (Treestar, San Carlos, CA, USA). At least 50,000 events were collected per sample. To verify leukocyte enrichment, selected samples were stained with anti-CD45 antibody (clone 9.4, Elabscience Biotechnology Inc., Houston, TX, USA). Thus, we defined the lymphocyte (Ly) gate by plotting FSC vs. SSC. To confirm the Ly gate in some stainings we added an additional tube with anti-CD45-APC antibody (clone HI30, Imunotools, Friesoythe, Germany) together with anti-CD3-FITC (clone UCHT-1, BD, San Jose, CA, USA) and γδ-PE (clone 11F2, BD, Taufkirchen, Germany) to confirm the Ly gate (especially in DMC suspensions). During the FACS analysis we excluded dead cells based on the unique scatter properties of viable and apoptotic cells. The apoptotic cells have high SSC/low FSC and viable cells have high FSC/low SSC properties [37]. By plotting γδTCR staining vs. NK-cell staining we defined the following cell populations: total γδ cells, total NK cells (CD56+), γδ + CD56-, γδ + CD56+, NK CD56^dim^ subset (γδ-CD56^dim^) and NK CD56^bright^ subset (γδ-CD56^bright^). The gating strategy for the data analyses is shown in Figure 2A. Identical gates were applied across samples. The CD107a expression was detected within each cell population as percentage of the positive cells and mean fluorescence intensity (MFI). MFI reflects the density of the CD107a molecules on the cell surface. An unstained or Fluorescence Minus One (FMO) control was used to identify and gate CD107-positive cells. MFI values were normalized to the corresponding FMO controls.

### 2.6. Multivariate and Clustering Analyses

To characterize global cytotoxic profiles across compartments and study groups, multivariate analyses were performed using CD107 expression parameters derived from all NK- and γδ T-cell populations. Dimensionality reduction and clustering were performed using the t-distributed stochastic neighbor embedding (tSNE) Cluster Explorer plugin implemented in FlowJo v10. This approach was used to visualize cellular heterogeneity and identify phenotypic relationships among NK-cell and γδ T-cell subsets based on marker-expression patterns. Principal component analysis (PCA) and linear discriminant analysis (LDA) were performed in R using scaled and centered CD107 expression variables. PCA was used to identify major sources of variation among samples, whereas LDA was used to evaluate discrimination between non-pregnant peripheral blood, pregnant peripheral blood, and decidual immune-cell populations. Generalized linear mixed-effects models (GLMMs) were applied to evaluate baseline differences in CD107 expression while accounting for donor-specific variability.

### 2.7. Immunocytochemistry

The expression of HLA-G was revealed by immunofluorescent staining of Sw71 and Sw71 *TW1*-KO cell monolayers, cultured on chamber slides (354118, FALCON, Corning, NY, USA). Cells were washed twice with PBS/0.1% BSA, fixed with ice-cold methanol for 5 min at −20 °C, and then washed twice. Then the cells were permeabilized with PBS/0.15% Triton X-100 and washed twice, and non-specific binding was blocked with Super Block in PBS/0.1% Tween-20 (1:10, AAA-015 ScyTek Laboratories, Logan, UT, USA). The cells were incubated with polyclonal rabbit anti-HLA-G (E-AB-18031, Elabscience, Houston, TX, USA), ON at 4 °C. After washing, the cells were incubated with secondary goat anti-rabbit AF488-conjugated antibody (E-AB-1055, Elabscience, Houston, TX, USA) for 2 h at room temperature. Appropriate negative controls omitting primary or secondary antibody were run in parallel. The slides were imaged with the confocal microscope Nikon AX (Nikon, Tokyo, Japan).

### 2.8. Statistical Analyses

All statistical analyses were performed in R (version 4.4.2). Data distribution was assessed using visual inspection and normality testing and paired or non-parametric tests were applied accordingly. For comparisons between two paired groups, paired Student’s *t*-tests were used for normally distributed data, whereas Wilcoxon signed-rank tests were applied when normality assumptions were not met. For comparisons involving three paired conditions (control, Sw71, and Sw71 *TW1*-KO), repeated-measures analysis of variance (RM-ANOVA) was used for normally distributed data, whereas Friedman tests were applied as the non-parametric alternative. When overall significance was detected, post hoc pairwise comparisons were performed using paired *t*-tests or Wilcoxon signed-rank tests, as appropriate. All tests were two-sided. Results are presented as the percentage of CD107a-positive cells and MFI of CD107a, which were analyzed independently. To account for multiple testing, *p*-values were adjusted using the Benjamini–Hochberg false discovery rate (FDR) procedure, generating corresponding q-values. FDR correction was applied separately within predefined families of analyses, including spontaneous degranulation comparisons, trophoblast-induced degranulation analyses, and post hoc pairwise comparisons. Results with *q* < 0.05 were considered statistically significant, while results with nominal *p* < 0.05 but *q* ≥ 0.05 were interpreted as trends. Data visualization was performed using paired dot plots with individual subject trajectories. Statistical significance is indicated in figures according to the corresponding *p*- or *q*-value thresholds.

## 3. Results

### 3.1. Baseline Differences in Spontaneous Degranulation Between Peripheral Blood and Decidual Immune Cells

The distribution of NK- and γδ T-cell subsets in peripheral blood and decidua is shown in Figure 2B. As expected, CD56^dim^ NK cells predominated in peripheral blood, whereas CD56^bright^ NK cells represented the dominant decidual NK-cell subset [6]. To characterize baseline degranulation across compartments, CD107 expression was analyzed using GLMM, PCA and LDA analyses. Several NK- and γδ T-cell subsets displayed differences in baseline CD107 MFI between peripheral blood and decidua (Figure 2C–E). LDA demonstrated clear separation between non-pregnant peripheral blood, pregnant peripheral blood, and decidual samples based on multivariate CD107 parameters (Figure 2C). Similarly, PCA identified a distinct clustering of decidual samples along the principal component space, whereas peripheral blood samples from pregnant and non-pregnant women clustered more closely together (Figure 2D). Heatmap analysis further demonstrated higher baseline CD107 MFI in several decidual NK- and γδ T-cell subsets compared with peripheral blood-derived populations (Figure 2E). Notably, decidual γδ T cells exhibited higher CD107 MFI than decidual NK cells, and CD107 expression was consistently higher in decidual γδ T cells than in their peripheral blood counterparts from both pregnant and non-pregnant women (Figure 2E).

**Figure 3:**
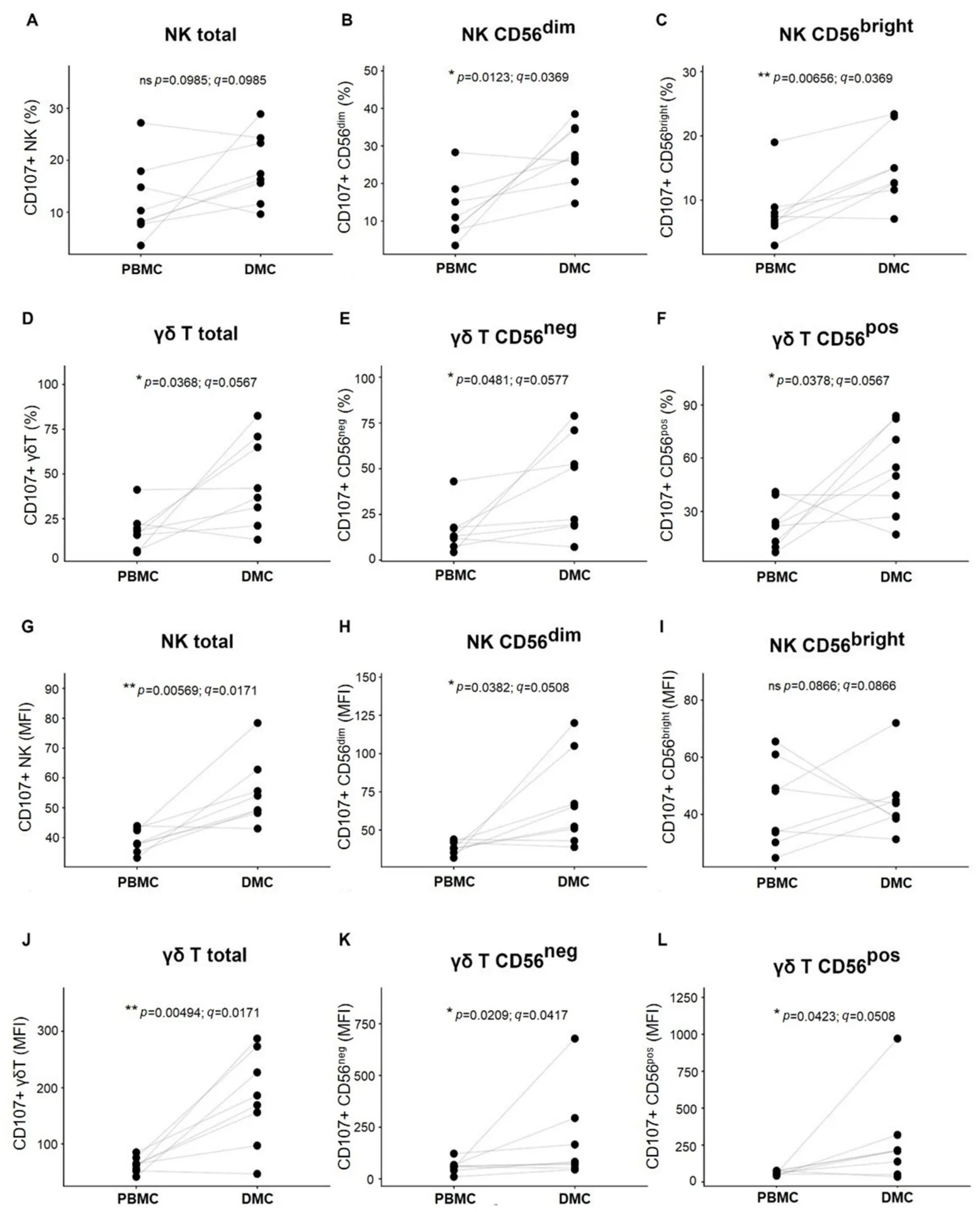
Baseline compartmentalization of spontaneous degranulation in NK- and γδ T-cell subsets between peripheral blood and decidua during early pregnancy shown by CD107a expression (as % and MFI). (**A**–**C**): Frequency (%) of CD107-positive total NK cells, CD56^dim^ NK cells, and CD56^bright^ NK cells in paired PBMCs and DMCs from pregnant women under baseline conditions. (**D**–**F**): Frequency (%) of CD107-positive total γδ T cells, CD56-neg γδ T cells, and CD56-pos γδ T cells in paired PBMC and DMC samples. (**G**–**I**): MFI of CD107 on total NK cells, CD56^dim^ NK cells, and CD56^bright^ NK cells in paired PBMC and DMC samples. (**J**–**L**): CD107 MFI on total γδ T cells, CD56-neg γδ T cells, and CD56-pos γδ T cells in paired PBMC and DMC samples. Each symbol represents an individual donor; lines connect matched PBMC and DMC samples from the same patient. Statistical significance was assessed using paired comparisons between compartments. *p*-values represent nominal significance, and q-values indicate Benjamini–Hochberg FDR-adjusted significance within each readout family.

In paired samples from pregnant women, decidual immune cells exhibited significantly higher spontaneous degranulation than matched peripheral blood cells (Figure 3). Both CD56^dim^ and CD56^bright^ decidual NK-cell subsets showed higher frequencies of CD107-positive cells compared with their peripheral blood counterparts (Figure 3A–C). A similar pattern was observed for γδ T cells. Total γδ T cells and both CD56-defined γδ T-cell subsets demonstrated increased frequencies of CD107-positive cells in decidua relative to peripheral blood (Figure 3D–F). Likewise, CD107 MFI was increased, indicating enhanced spontaneous degranulation within the decidual compartment in both NK cells (Figure 3G–I) and γδ T cells (Figure 3J–L). Collectively, these findings demonstrate compartment-specific differences in baseline degranulation, with decidual NK and γδ T cells exhibiting higher spontaneous CD107 expression than peripheral blood-derived cells.

### 3.2. Baseline Degranulation of NK- and γδ T-Cell Subsets

Spontaneous degranulation was next compared between NK cells and γδT cells within the same donor samples (Table S1). In pregnant women, γδT cells exhibited significantly higher spontaneous degranulation than NK cells in both peripheral blood and decidua. This difference was reflected by higher CD107 MFI in peripheral blood and by both increased CD107 MFI and higher frequencies of CD107-positive cells in decidua (Table S1). No significant differences in spontaneous degranulation were detected between CD56+ and CD56- γδ T-cell subsets in either compartment. In contrast, decidual NK-cell subsets differed significantly, with CD56^dim^ NK cells displaying higher frequencies of CD107-positive cells than CD56^bright^ NK cells (Table S1). This difference was not observed in peripheral blood from pregnant women and did not remain significant after correction for multiple testing in non-pregnant controls.

### 3.3. Effect of EVT-Derived Signals on NK-Cell Degranulation

**Figure 4:**
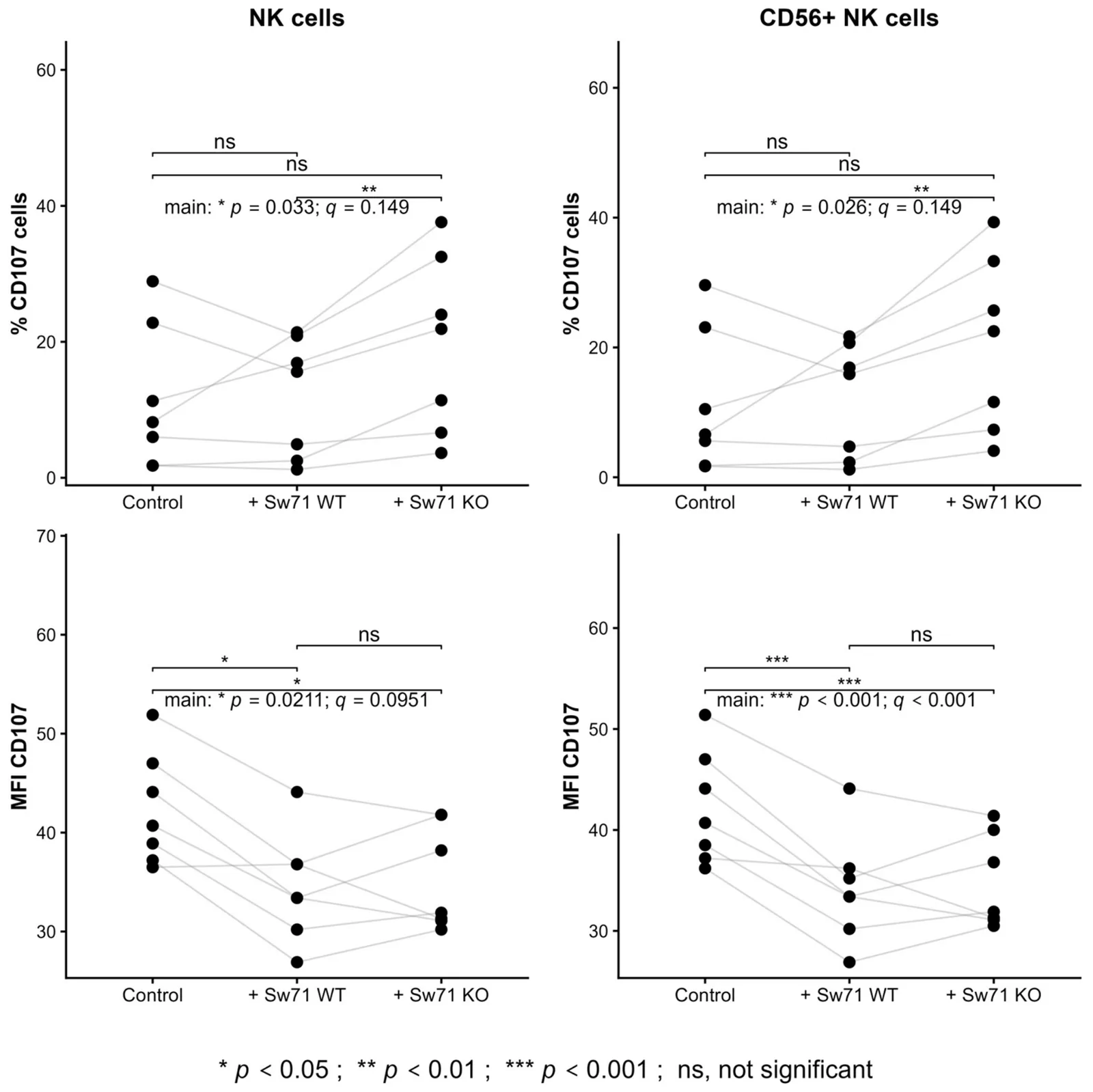
NK-cell degranulation within PBMCs from non-pregnant women co-cultured with trophoblast cells. (**Up**)—A trend of an increased number of CD107-positive cells within total NK cells and their CD56^dim^ subset in PBMCs of non-pregnant women, co-cultured with Sw71 TW1-KO EVT-like cells. (**Down**)—CD56^dim^ NK cells showed significantly reduced CD107 MFI after exposure to Sw71 trophoblasts regardless of their HLA-G/C intensity.

The influence of trophoblast-derived signals on NK-cell degranulation was assessed using co-culture with WT Sw71 EVT-like cells and with the Sw71 TW1-KO clone with reduced expression of HLA-G and HLA-C [29] (Figure S1). In PBMCs from pregnant women, co-culture with either Sw71 or Sw71 *TW1*-KO trophoblasts did not significantly alter the degranulation in total NK cells or in CD56-defined NK-cell subsets, regardless of the frequency of CD107-positive cells or CD107 MFI (Table S2). In DMCs, co-culture with Sw71 *TW1*-KO cells was associated with increased frequencies of CD107-positive CD56^dim^ NK cells compared with control conditions; however, this effect did not remain significant following FDR correction (Table S2). In contrast, PBMCs from non-pregnant women exhibited significant modulation of NK-cell degranulation following trophoblast co-culture. There was a trend of an increased number of CD107-positive cells among total NK cells and their CD56^dim^ subset in PBMCs of non-pregnant women, following co-culture with the Sw71 TW1-KO cells. CD56^dim^ NK cells showed reduced CD107 MFI after exposure to Sw71 trophoblasts, and this effect remained significant after correction for multiple testing (Table S2, Figure 4). A comparable reduction was observed following co-culture with Sw71 *TW1*-KO cells.

### 3.4. Effect of EVT-Derived Signals on γδ T-Cell Degranulation

**Figure 5:**
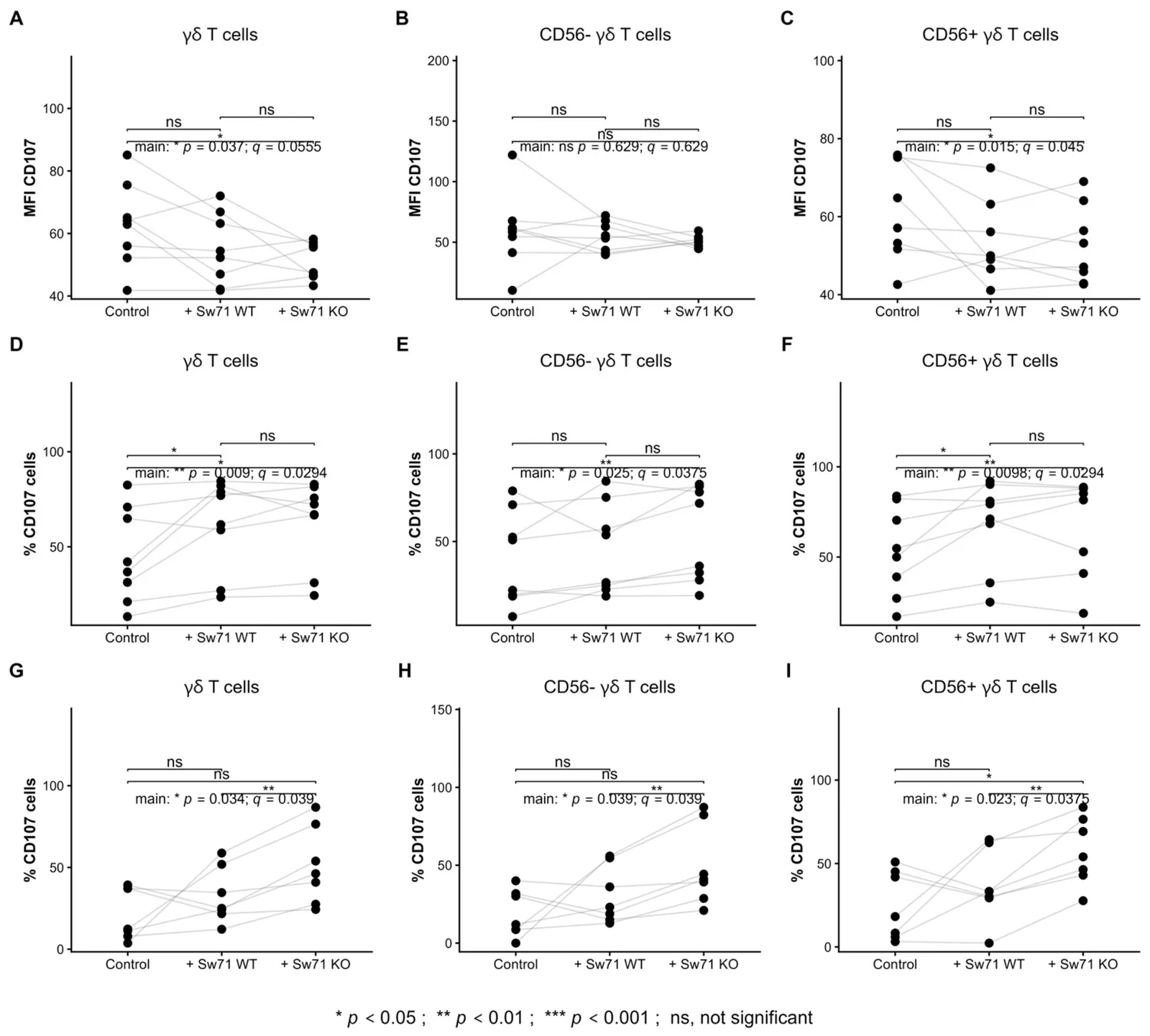
Effect of trophoblast co-culture on γδT-cell degranulation. (**A**–**C**): Note the trend of CD107-positive cells decreasing (MFI and frequency) on total γδ T cells and their CD56+ subset when PBMCs from pregnant women were co-cultured with trophoblasts, regardless of the intensity of HLA-G and -C expression. (**D**–**F**): Increased frequency of CD107-positive CD56^+^ γδ T cells in DMCs from pregnant women following co-culture with Sw71 or Sw71 *TW1*-KO trophoblast cells. (**G**–**I**): γδ T cells and both CD56-based subsets showed a trend to degranulate more when co-cultured with WT EVTs and even more when co-cultured with *TW1*-KO EVTs. Each symbol represents an individual donor; lines connect paired samples from the same donor across conditions. Summary data are shown as mean ± bootstrap 95% confidence intervals. Data are presented as mean ± SD. The main *p*-values derive from repeated-measures ANOVA (RM_ANOVA) or Friedman tests comparing control, Sw71, and Sw71 *TW1*-KO conditions within matched donor samples. q-values were calculated using Benjamini–Hochberg FDR correction. PBMCs = peripheral blood mononuclear cells; DMCs = decidual mononuclear cells.

In PBMCs from pregnant women, co-culture with EVT-like trophoblasts was associated with reduced CD107 MFI in total γδ T cells and in the CD56+ γδ T-cell subset. Similar reductions were observed in the frequency of CD107-positive CD56+ γδ T cells. These effects, however, did not remain significant following correction for multiple testing. No consistent changes were detected in the CD56- γδ T-cell subset (Table S3, Figure 5A–C). In decidual samples, trophoblast co-culture significantly increased the frequency of CD56+ γδ T cells, as confirmed with FDR correction (Table S3, Figure 5D–F). Similar trends were observed for CD56- γδ T cells but did not retain significance after multiple-testing correction. In PBMCs from non-pregnant women, trophoblast co-culture was associated with increased frequencies of CD107-positive γδ T cells and their CD56-defined subsets, although these effects generally did not remain significant after correction for multiple testing (Table S3, Figure 5G–I).

Taken together, trophoblast-derived signals exerted limited effects on NK-cell degranulation during pregnancy but significantly modulated decidual CD56+ γδ T-cell degranulation.

## 4. Discussion

This study provides a functional characterization of NK- and γδ T-cell subset degranulation across peripheral blood and decidual compartments during early pregnancy and examines how these responses are influenced by trophoblast-derived signals. Using paired maternal samples and EVT-like cell models differing in HLA-G/HLA-C expression, we demonstrate that cytotoxic regulation during early pregnancy is highly compartmentalized and cell-type-specific. Our findings reveal a distinct decidual cytotoxic landscape characterized by enhanced baseline degranulation of both NK and γδ T cells, differential responsiveness of these populations to trophoblast-derived signals, and evidence that pregnancy-associated immune adaptation alters the sensitivity of cytotoxic cells to trophoblast-mediated regulation. The maternal–fetal interface represents a unique immunological environment in which fetal tolerance must coexist with protection against infection and abnormal cellular transformation [23,38]. Such an intricate microenvironment presupposes tissue-specialized immune-cell populations, distinct from their peripheral counterparts in phenotype and effector functions. Consistent with this concept, we observed significantly higher spontaneous degranulation of decidual NK and γδ T cells compared with their matched peripheral blood counterparts. These findings suggest that early pregnancy is associated with a locally primed cytotoxic immune landscape in the decidua. Although dNK cells are traditionally regarded as predominantly regulatory and cytokine-producing cells [39], our data indicate that both CD56^dim^ and CD56^bright^ decidual NK-cell subsets retain measurable cytotoxic readiness. The two major NK-cell subsets differ in their responsiveness, proliferative response to IL-2, intrinsic cytotoxic capacity, NKR repertoire, and adhesion molecule expression [40]. Notably, CD56^dim^ decidual NK cells exhibited greater degranulation than CD56^bright^ cells, consistent with their more differentiated phenotype and established cytotoxic potential [41,42,43,44], whereas CD56^bright^ NK cells are more proficient in cytokine production [45], and express low levels of perforin and high levels of the CD94/NKG2 receptor and adhesion-mediating L-selectin [46]. Indeed, we found that dNK cells displayed clear subset-specific differences in their cytotoxicity: as expected, CD56^dim^ NK cells were more prone to degranulate than CD56^bright^ NK cells.

Similarly, decidual γδ T-cell subsets displayed enhanced degranulation (at both the population and per-cell levels) compared with circulating counterparts, with the CD56+ subset exhibiting the strongest cytotoxic profile. Peripheral blood γδ T cells exhibited comparable degranulation rates in both pregnant and non-pregnant women. These data confirm our previous data with different cohorts of pregnant and non-pregnant women [20]. Regarding the comparison between the two cytotoxic populations in the pregnant women, we revealed that at baseline γδ T cells compared to NK cells showed higher spontaneous degranulation (per population and cell level) in both locations—decidua and blood. However, no such difference was observed in the blood of non-pregnant women. Together, these observations support the concept that decidual cytotoxic lymphocytes exist in a state of controlled activation that enables rapid responses to local challenges while maintaining pregnancy-associated tolerance.

An additional finding of this study is the differential responsiveness of NK and γδ T cells to EVT-derived signals. To address this question, we employed the EVT-like Sw71 cell line and its *TWIST1*-deficient derivative, a previously characterized clone reported to exhibit reduced expression of HLA-G and HLA-C and impaired invasive properties [29,33]. EVT invasion is only observed in great apes [47], which limits the use of animal models. Primary human EVTs are non-proliferating difficult-to-culture cells [48], and their access raises ethical and practical concerns. In order to advance our knowledge about maternal cytotoxic cells, EVT interaction is essential to apply to relevant trophoblast cell line experimental models. Previous studies have utilized virus-transfected (HTR-8/SVneo) or choriocarcinoma-derived (BeWo) cell lines, which do not accurately represent the characteristics of normal invasive EVTs. Together with human choriocarcinoma cell line JAr, these do not express HLA molecules [49]. The EVT model used in this study enabled us to investigate how alterations in trophoblast phenotype influence cytotoxic-cell function while overcoming many of the practical limitations associated with primary EVT cultures. The normal human immortalized Sw71 EVT-like cell line effectively bridges the gap between limited primary tissue availability and the shortcomings of choriocarcinoma-derived trophoblast models. We have proved that Sw71 cells express key EVT identity markers, such as CK7, Vim, and HLA-G, HLA-C, and HLA-F molecules at levels comparable to primary EVTs [31,50]. Sw71 EVT-like cells demonstrate the ability to form blastocyst-like structures (spheroids, BLSs) that structurally mirror primary cytotrophoblast spheroids, featuring the capacity for outward EVT migration and invasion between endometrial stromal cells and ECM [30,51,52]. In parallel, we use a clone of genetically modified Sw71 *TW1*-KO EVT-like cells proven to have downregulated around 1140 genes, including the MHC class I molecules HLA-G and HLA-C, compared to WT Sw71 EVT-like cells [29]. Moreover, Sw71 *TW1*-KO EVT-like cells have impaired migration and shallow/blocked invasion [33]. Here, we showed that NK cells from pregnant women showed little or no change in degranulation following co-culture with EVT-like cells, regardless of trophoblast HLA-G/HLA-C expression. This finding was observed in both peripheral blood and decidual compartments and suggests that pregnancy-associated adaptation may establish a relatively stable functional state in which NK-cell responsiveness to trophoblast-derived inhibitory signals is already optimized (NK licensing) [53,54]. Such pre-adaptation may serve to stabilize immune tolerance during early pregnancy while preserving compartment-specific regulatory control. In contrast, peripheral NK cells from non-pregnant women displayed marked suppression of CD56^dim^ NK-cell degranulation following exposure to EVT-like cells. The observation that this inhibitory effect occurred in the presence of both wild-type and *TWIST1*-deficient trophoblasts suggests that mechanisms beyond HLA-G/HLA-C-mediated interactions contribute to trophoblast-induced regulation of circulating NK cells and will require direct mechanistic investigation.

Collectively, these findings support the notion that trophoblast-associated signals exert stronger modulatory effects on non-pregnancy-adapted NK cells than on NK cells already conditioned by the pregnant state. Our findings about unresponsivess of NK cells from pregnant women to trophoblasts correspond to the previously published data about the lack of healthy trophoblast killing by dNK cells [38,55]. However, dNK cells promptly become capable of attacking fetal and maternal tissues during infection and inflammation [24,56].

γδ T cells demonstrated a distinct and more nuanced EVT-response profile. Rather than exhibiting global suppression, trophoblast co-culture selectively influenced specific γδ T-cell subsets, most notably CD56^+^ γδ T cells. Whereas peripheral γδT cells showed limited responsiveness to trophoblast co-culture, decidual CD56+ γδ T cells exhibited enhanced degranulation following exposure to EVT-like cells, particularly when trophoblast HLA-G/HLA-C expression was reduced. Although the differences in HLA-G/HLA-C expression that contribute to this response cannot be directly established from the present data, sensitivity of γδ T cells to local alterations in trophoblast immune signaling could be suggested. These observations imply that γδ T cells may be more sensitive than NK cells to local changes in trophoblast immune phenotype. Given the ability of γδ T cells to integrate signals from both γδ T-cell receptors and NK-associated receptors, such responsiveness may allow these cells to function as highly adaptable sensors of tissue stress and trophoblast integrity. Although the molecular pathways underlying these effects were not directly examined in the present study, receptor systems involving KIRs, CD94/NKG2A-HLA-E interactions, immune checkpoint pathways, or other trophoblast-derived regulatory signals may contribute to the observed functional differences and warrant future investigation. The combined findings of enhanced baseline decidual cytotoxicity and selective responsiveness to trophoblast-derived cues support a model in which decidual cytotoxic lymphocytes are not functionally silenced but instead exist in a tightly regulated state of readiness. Such a state is biologically consistent with the physiological requirements of early pregnancy, including tissue remodeling, vascular adaptation, regulation of trophoblast invasion, and protection against pathogens. The ability to rapidly transition between tolerance and effector function may be particularly important at the maternal–fetal interface, where excessive immune activation can threaten pregnancy, yet insufficient immune surveillance may compromise tissue integrity and host defense. Several limitations should be considered when interpreting these findings. The sample size was necessarily constrained by the availability of paired decidual and peripheral blood samples and by the complexity of functional co-culture experiments. In addition, the study focused on degranulation as a functional readout and did not directly investigate the molecular mechanisms responsible for the observed responses. Furthermore, although the EVT-like models used here provide important experimental advantages and recapitulate key trophoblast characteristics, they cannot fully reproduce the complexity of the in vivo decidual microenvironment. Nevertheless, the paired analytical approach, consistency of effect directions across donors, and biologically coherent response patterns across cell types and compartments lend support to the robustness of the observed trends.

## 5. Conclusions

In conclusion, our results demonstrate that the regulation of immune-cell degranulation during early pregnancy is highly compartmentalized and differs substantially between NK- and γδ T-cell populations. Decidual cytotoxic cells exhibit enhanced baseline functional readiness, while their responsiveness to trophoblast-derived signals is shaped by both pregnancy status and tissue localization. These findings provide new insight into the mechanisms that balance immune tolerance and immune surveillance at the maternal–fetal interface and establish a framework for future studies investigating immune dysregulation in pregnancy complications such as recurrent pregnancy loss and preeclampsia.

## Data Availability

The datasets generated and analyzed during the current study are available from the corresponding author upon reasonable request.

## Supplementary Materials

The following supporting information can be downloaded at https://www.mdpi.com/article/10.3390/cells15181691/s1. Table S1. Differential spontaneous degranulation of NK- versus γδ T-cell subsets in peripheral blood and decidua of pregnant and non-pregnant women. Baseline spontaneous degranulation was compared between NK-cell and γδ T-cell populations within the same donor samples. Values are presented as mean ± SD. Statistical significance was assessed using paired *t*-tests or Wilcoxon signed-rank tests depending on the normality of paired differences. q-values were calculated using Benjamini–Hochberg FDR correction. Table S2. Effect of trophoblast co-culture on NK-cell degranulation in peripheral blood and decidual mononuclear cells. Descriptive statistics and repeated-measures analyses for CD107 degranulation responses of total NK cells and their subsets CD56^dim^ and CD56^bright^ cells following co-culture with Sw71 or Sw71 TW1-KO trophoblast cells under control and trophoblast-exposed conditions in DMCs or PBMCs from pregnant and non-pregnant women. Data are presented as mean ± SD. Multiple-testing correction was performed using the Benjamini–Hochberg FDR method. Table S3. Effect of trophoblast co-culture on γδT-cell degranulation. Data are presented as mean ± SD. The main *p*-values derive from repeated-measures ANOVA (RM_ANOVA) or Friedman tests comparing control, Sw71, and Sw71 TW1-KO conditions within matched donor samples. q-values were calculated using Benjamini–Hochberg FDR correction. Figure S1. Differential intensity of HLA-G and HLA-C expression by the Sw71 EVT-like cells and their genetically modified counterparts Sw71-TW1-KO cells.

## Abbreviations

The following abbreviations are used in this manuscript:

CTB	Cytotrophoblast
DMCs	Decidual mononuclear cells
dNK	Decidual NK cells
EP	Early pregnancy
EMT	Epithelial–mesenchymal transition
EVTs	Extravillous trophoblasts
FDR	False discovery rate
FMO	Fluorescence Minus One
GLMMs	Generalized linear mixed-effects models
iKIRs	Inhibitory killer-cell immunoglobulin-like receptors
KIRs	Killer-cell immunoglobulin-like receptors
LDA	Linear discriminant analysis
MHC	Major histocompatibility complex
MFI	Mean fluorescence intensity
NK	Natural killer cells
NP	Non-pregnant
PBMCs	Peripheral blood mononuclear cells
PCA	Principal component analysis
RM-ANOVA	Repeated-measures analysis of variance
SYN	Syncytiotrophoblast
tSNE	t-distributed stochastic neighbor embedding
