# Supplementary material for: Compartmentalized and Differential Regulation of NK- and γδ T-Cell Degranulation by Trophoblast-Derived Signals During Early Pregnancy
Source: Cells. 2026 Sep 17;15(18):1691. doi: 10.3390/cells15181691 (PMC13604995; doi:10.3390/cells15181691)
Supplement: Supplementary file 1 [file cells-15-01691-s001.zip › Tables S1-S2-S3.pdf]

**Table S1. Differential spontaneous degranulation of NK and γδ T-cell subsets in peripheral blood and decidua of pregnant and non-pregnant women.** Baseline spontaneous degranulation was compared between NK-cell and γδ T-cell populations within the same donor samples. Values are presented as mean ± SD. Statistical significance was assessed using paired t-tests or Wilcoxon signed-rank tests depending on normality of paired differences. q-values were calculated using Benjamini–Hochberg false discovery rate correction.

| Comparison | Group | Compartment | Readout | n | Population A (mean ± SD) | Population B (mean ± SD) | Test | p | q | Interpretation |
|---|---|---|---|---|---|---|---|---|---|---|
| γδ T vs total NK | Pregnant | PBMC | CD107+ cells (%) | 8 | 18.34 ± 10.82 | 12.20 ± 7.53 | Paired t-test | 0.083 | 0.141 | Not significant |
| γδ T vs total NK | Pregnant | PBMC | CD107 MFI | 8 | 62.84 ± 13.44 | 38.94 ± 3.88 | Paired t-test | 0.0024 | 0.0109 | FDR-significant |
| γδ T CD56+ vs CD56− | Pregnant | PBMC | CD107+ cells (%) | 8 | 22.32 ± 12.64 | 16.00 ± 11.82 | Wilcoxon paired | 0.031 | 0.095 | Nominal effect / trend |
| γδ T CD56+ vs CD56− | Pregnant | PBMC | CD107 MFI | 8 | 62.02 ± 12.80 | 59.59 ± 31.08 | Paired t-test | 0.629 | 0.734 | Not significant |
| NK CD56bright vs CD56dim | Pregnant | PBMC | CD107+ cells (%) | 8 | 8.19 ± 4.71 | 12.51 ± 7.92 | Paired t-test | 0.118 | 0.177 | Not significant |
| NK CD56bright vs CD56dim | Pregnant | PBMC | CD107 MFI | 8 | 43.36 ± 14.89 | 38.65 ± 4.01 | Paired t-test | 0.327 | 0.491 | Not significant |
| γδ T vs total NK | Pregnant | DMC | CD107+ cells (%) | 8 | 45.36 ± 24.82 | 18.38 ± 6.61 | Paired t-test | 0.012 | 0.036 | FDR-significant |
| γδ T vs total NK | Pregnant | DMC | CD107 MFI | 8 | 180.25 ± 82.49 | 55.04 ± 11.14 | Wilcoxon paired | 0.0026 | 0.0109 | FDR-significant |
| γδ T CD56+ vs CD56− | Pregnant | DMC | CD107+ cells (%) | 8 | 53.04 ± 24.72 | 40.12 ± 26.77 | Wilcoxon paired | 0.041 | 0.095 | Nominal effect / trend |
| γδ T CD56+ vs CD56− | Pregnant | DMC | CD107 MFI | 8 | 269.01 ± 298.62 | 182.81 ± 216.59 | Wilcoxon paired | 0.674 | 0.734 | Not significant |

| | | | | | | | | | | |
|---|---|---|---|---|---|---|---|---|---|---|
| NK CD56bright vs CD56dim | Pregnant | DMC | CD107+ cells (%) | 8 | 15.05 ± 5.61 | 27.88 ± 7.90 | Paired t-test | 0.00094 | 0.0048 | FDR-significant |
| NK CD56bright vs CD56dim | Pregnant | DMC | CD107 MFI | 8 | 44.51 ± 12.10 | 67.83 ± 29.52 | Paired t-test | 0.071 | 0.141 | Not significant |
| γδ T vs total NK | NonPregnant | PBMC | CD107+ cells (%) | 7 | 21.26 ± 15.79 | 11.54 ± 10.49 | Paired t-test | 0.083 | 0.141 | Not significant |
| γδ T vs total NK | NonPregnant | PBMC | CD107 MFI | 7 | 74.67 ± 32.78 | 42.33 ± 5.65 | Paired t-test | 0.040 | 0.095 | Nominal effect / trend |
| γδ T CD56+ vs CD56− | NonPregnant | PBMC | CD107+ cells (%) | 7 | 24.79 ± 20.51 | 18.77 ± 15.01 | Paired t-test | 0.427 | 0.427 | Not significant |
| γδ T CD56+ vs CD56− | NonPregnant | PBMC | CD107 MFI | 7 | 70.36 ± 34.53 | 53.66 ± 28.16 | Paired t-test | 0.212 | 0.318 | Not significant |
| NK CD56bright vs CD56dim | NonPregnant | PBMC | CD107+ cells (%) | 7 | 12.43 ± 9.33 | 11.27 ± 10.89 | Wilcoxon paired | 0.312 | 0.312 | Not significant |
| NK CD56bright vs CD56dim | NonPregnant | PBMC | CD107 MFI | 7 | 42.20 ± 9.38 | 42.16 ± 5.60 | Paired t-test | 0.984 | 0.984 | Not significant |

**Table S2. Effect of trophoblast co-culture on NK-cell degranulation in peripheral blood and decidual mononuclear cells.** Descriptive statistics and repeated-measures analyses for CD107 degranulation responses of total NK cells, CD56^dim^ NK cells, and CD56^bright^ NK cells following co-culture with Sw71 or Sw71-TwKO trophoblast cells under control and trophoblast-exposed conditions in peripheral blood mononuclear cells (PBMC) and decidual mononuclear cells (DMC) from pregnant and non-pregnant women. Data are presented as mean ± standard deviation (SD). Multiple-testing correction was performed using the Benjamini–Hochberg false discovery rate method.

| Group | Compartment | Subset | Readout | n | Control | Sw71 | Sw71_Tw1-KO | Test | Main p | Main q | Interpretation |
|---|---|---|---|---|---|---|---|---|---|---|---|
| NonPregnant | PBMC | CD56bright NK cells | CD107 MFI | 7 | 42.2 ± 9.38 | 49.83 ± 37.12 | 50.59 ± 37.97 | Friedman | 0.651 | 0.651 | not significant |
| NonPregnant | PBMC | CD56bright NK cells | CD107+ cells (%) | 7 | 12.43 ± 9.33 | 14.11 ± 15.32 | 19.69 ± 16.53 | Friedman | 0.156 | 0.351 | not significant |
| NonPregnant | PBMC | CD56dim NK cells | CD107 MFI | 7 | 42.16 ± 5.60 | 34.20 ± 5.38 | 34.71 ± 4.61 | RM_ANOVA | 0.0000284 | 0.000256 | FDR-significant main condition effect |
| NonPregnant | PBMC | CD56dim NK cells | CD107+ cells (%) | 7 | 11.27 ± 10.89 | 11.92 ± 8.87 | 20.54 ± 13.36 | RM_ANOVA | 0.026 | 0.149 | nominal main condition effect / trend |
| NonPregnant | PBMC | Total NK cells | CD107 MFI | 7 | 42.33 ± 5.65 | 34.51 ± 5.50 | 35.19 ± 5.23 | Friedman | 0.0211 | 0.0951 | nominal main condition effect / trend |
| NonPregnant | PBMC | Total NK cells | CD107+ cells (%) | 7 | 11.54 ± 10.49 | 11.92 ± 8.76 | 19.67 ± 12.94 | RM_ANOVA | 0.033 | 0.149 | nominal main condition effect / trend |
| Pregnant | DMC | CD56bright | CD107 | 8 | 44.51 ± | 55.65 | 53.33 ± | Friedman | 0.368 | 0.573 | not significant |

| | | | | | | | | | | | |
|---|---|---|---|---|---|---|---|---|---|---|---|
| | | NK cells | MFI | | 12.10 | ± 20.37 | 19.64 | | | | |
| Pregnant | DMC | CD56bright NK cells | CD107+ cells (%) | 8 | 15.04 ± 5.61 | 14.20 ± 6.65 | 14.93 ± 7.09 | RM_ANOVA | 0.739 | 0.739 | not significant |
| Pregnant | DMC | CD56dim NK cells | CD107 MFI | 8 | 67.83 ± 29.52 | 75.34 ± 30.24 | 81.44 ± 37.89 | RM_ANOVA | 0.446 | 0.573 | not significant |
| Pregnant | DMC | CD56dim NK cells | CD107+ cells (%) | 8 | 27.88 ± 7.90 | 31.23 ± 10.68 | 34.19 ± 13.30 | RM_ANOVA | 0.096 | 0.288 | not significant |
| Pregnant | DMC | Total NK cells | CD107 MFI | 8 | 55.04 ± 11.14 | 62.05 ± 25.24 | 66.40 ± 25.43 | RM_ANOVA | 0.380 | 0.573 | not significant |
| Pregnant | DMC | Total NK cells | CD107+ cells (%) | 8 | 18.38 ± 6.61 | 19.59 ± 7.53 | 20.36 ± 9.99 | RM_ANOVA | 0.489 | 0.629 | not significant |
| Pregnant | PBMC | CD56bright NK cells | CD107 MFI | 8 | 43.36 ± 14.89 | 37.14 ± 11.71 | 41.11 ± 6.82 | RM_ANOVA | 0.570 | 0.641 | not significant |
| Pregnant | PBMC | CD56bright NK cells | CD107+ cells (%) | 8 | 8.19 ± 4.71 | 9.25 ± 7.11 | 8.11 ± 5.53 | RM_ANOVA | 0.601 | 0.676 | not significant |
| Pregnant | PBMC | CD56dim NK cells | CD107 MFI | 8 | 38.65 ± 4.01 | 38.96 ± 4.21 | 36.54 ± 6.19 | RM_ANOVA | 0.236 | 0.573 | not significant |
| Pregnant | PBMC | CD56dim NK cells | CD107+ cells (%) | 8 | 12.51 ± 7.92 | 9.99 ± 7.85 | 10.23 ± 4.68 | RM_ANOVA | 0.265 | 0.441 | not significant |
| Pregnant | PBMC | Total NK cells | CD107 MFI | 8 | 38.94 ± 3.88 | 39.30 ± 3.99 | 37.30 ± 6.21 | RM_ANOVA | 0.388 | 0.573 | not significant |

| | | | | | | | | | | | |
|---|---|---|---|---|---|---|---|---|---|---|---|
| Pregnant | PBMC | Total NK cells | CD107+ cells (%) | 8 | 12.20 ± 7.53 | 9.90 ± 7.51 | 10.00 ± 4.78 | RM_ANOVA | 0.294 | 0.441 | not significant |

**Table S3. Effect of trophoblast co-culture on γδ T-cell degranulation.** Data are presented as mean ± SD. Main p values derive from repeated-measures ANOVA (RM_ANOVA) or Friedman tests comparing Control, Sw71, and Sw71_TwKO conditions within matched donor samples. q values were calculated using Benjamini–Hochberg false discovery rate correction. PBMC = peripheral blood mononuclear cells; DMC = decidual mononuclear cells.

| Group | Compartment | Subset | Readout | n | Control | Sw71 | Sw71_Tw1-KO | Test | Main p | Main q | Interpretation |
|---|---|---|---|---|---|---|---|---|---|---|---|
| NonPregnant | PBMC | CD56+ γδ T cells | CD107 MFI | 7 | 70.36 ± 34.53 | 97.03 ± 90.16 | 58.17 ± 10.24 | Friedman | 0.772 | 0.868 | not significant |
| NonPregnant | PBMC | CD56+ γδ T cells | CD107+ cells (%) | 7 | 24.79 ± 20.51 | 36.46 ± 21.33 | 57.21 ± 20.07 | RM_ANOVA | 0.023 | 0.0501 | nominal main condition effect / trend |
| NonPregnant | PBMC | CD56− γδ T cells | CD107 MFI | 7 | 53.66 ± 28.16 | 44.39 ± 4.98 | 50.31 ± 3.25 | RM_ANOVA | 0.573 | 0.809 | not significant |
| NonPregnant | PBMC | CD56− | CD107+ | 7 | 18.77 ± | 30.91 ± | 49.10 ± | RM_ANOV | 0.039 | 0.0501 | nominal main |

| | | | | | | | | | | | |
|---|---|---|---|---|---|---|---|---|---|---|---|
| | | γδ T cells | cells (%) | | 15.01 | 18.29 | 25.65 | A | | | condition effect / trend |
| NonPregnant | PBMC | Total γδ T cells | CD107 MFI | 7 | 74.67 ± 32.78 | 47.69 ± 7.65 | 53.50 ± 2.23 | Friedman | 0.0498 | 0.149 | nominal main condition effect / trend |
| NonPregnant | PBMC | Total γδ T cells | CD107+ cells (%) | 7 | 21.26 ± 15.79 | 32.63 ± 16.96 | 50.87 ± 23.56 | RM_ANOVA | 0.034 | 0.0501 | nominal main condition effect / trend |
| Pregnant | DMC | CD56+ γδ T cells | CD107 MFI | 8 | 269.01 ± 298.62 | 199.74 ± 107.09 | 255.15 ± 206.80 | Friedman | 0.607 | 0.809 | not significant |
| Pregnant | DMC | CD56+ γδ T cells | CD107+ cells (%) | 8 | 53.04 ± 24.72 | 67.86 ± 24.70 | 68.01 ± 26.97 | Friedman | 0.0098 | 0.0441 | FDR-significant main condition effect |
| Pregnant | DMC | CD56− γδ T cells | CD107 MFI | 8 | 182.81 ± 216.59 | 179.12 ± 163.28 | 118.10 ± 64.87 | Friedman | 0.882 | 0.882 | not significant |
| Pregnant | DMC | CD56− γδ T cells | CD107+ cells (%) | 8 | 40.12 ± 26.77 | 45.45 ± 25.66 | 53.70 ± 27.17 | RM_ANOVA | 0.025 | 0.0501 | nominal main condition effect / trend |
| Pregnant | DMC | Total γδ T cells | CD107 MFI | 8 | 180.25 ± 82.49 | 208.85 ± 156.54 | 219.52 ± 189.01 | Friedman | 0.607 | 0.809 | not significant |
| Pregnant | DMC | Total γδ T cells | CD107+ cells (%) | 8 | 45.36 ± 24.82 | 61.69 ± 24.30 | 62.73 ± 22.45 | RM_ANOVA | 0.009 | 0.0441 | FDR-significant main condition effect |
| Pregnant | PBMC | CD56+ γδ T cells | CD107 MFI | 8 | 62.02 ± 12.80 | 53.45 ± 10.11 | 52.66 ± 9.88 | RM_ANOVA | 0.015 | 0.135 | nominal main condition effect / trend |
| Pregnant | PBMC | CD56+ | CD107+ | 8 | 22.32 ± | 14.11 ± | 20.18 ± | Friedman | 0.0302 | 0.0501 | nominal main |

| | | | | | | | | | | | |
|---|---|---|---|---|---|---|---|---|---|---|---|
| | | γδ T cells | cells (%) | | 12.64 | 7.04 | 15.50 | | | | condition effect / trend |
| Pregnant | PBMC | CD56− γδ T cells | CD107 MFI | 8 | 59.59 ± 31.08 | 54.44 ± 12.42 | 50.58 ± 4.72 | RM_ANOVA | 0.629 | 0.809 | not significant |
| Pregnant | PBMC | CD56− γδ T cells | CD107+ cells (%) | 8 | 16.00 ± 11.82 | 10.93 ± 3.50 | 12.61 ± 7.39 | Friedman | 0.284 | 0.284 | not significant |
| Pregnant | PBMC | Total γδ T cells | CD107 MFI | 8 | 62.84 ± 13.44 | 54.98 ± 11.39 | 51.36 ± 5.96 | RM_ANOVA | 0.037 | 0.149 | nominal main condition effect / trend |
| Pregnant | PBMC | Total γδ T cells | CD107+ cells (%) | 8 | 18.34 ± 10.82 | 12.05 ± 4.25 | 14.40 ± 8.28 | RM_ANOVA | 0.081 | 0.0911 | not significant |
